# Supplementary material for: Nde1 promotes Lis1-mediated activation of dynein
Source: Nat Commun. 2023 Nov 9;14:7221. doi: 10.1038/s41467-023-42907-x (PMC10632352; doi:10.1038/s41467-023-42907-x)
Supplement: Supplementary file 1 — Supplementary Information [file 41467_2023_42907_MOESM1_ESM.pdf]

Nde1 Promotes Lis1-Mediated Activation of Dynein

Yuanchang Zhao[1,2], Sena Oten[2], Ahmet Yildiz[1,2,3*]

[1] Physics Department, University of California, Berkeley, CA, USA, 94709

[2] Department of Molecular and Cell Biology, University of California, Berkeley, CA, USA, 94709

[3] Biophysics Graduate Group, University of California, Berkeley, CA, USA, 94709

[*]Correspondence to: Ahmet Yildiz (yildiz@berkeley.edu)

Supplementary Information

1. Supplementary Tables

Construct	Vector	Source	Figures
GFP-DYNH1C1-SNAPf-IC2C-LIC2-ROBL1-TCTEX1-LC8	pOmniBac-pIDC	Carter Lab	F1b-e,g, F2g,h, F3e,f, F4, F5, F6f-i, S1, S3, S5, S6f, S8, S9
SNAPf-DYNH1C1-IC2C-LIC2-ROBL1-TCTEX1-LC8	pOmniBac-pIDC	Schlager et al. 2014[9]	F2d, e, F3c,d, F6d, S4, S3a,
SNAPf-DYNH1C1$^{R1567E, K1610E}$-IC2CLIC2-ROBL1-TCTEX1-LC8	pOmniBac-pIDC	Zhang et al. 2017[6]	F1f,g, F4e-g, F5b-d, S2, S6, S8
BICDR1-SNAPf	pOmniBac	Urnavicius et al., 2018[73]	F1, F2g,h, F3e,f, F4e-l, F5, F6f-i, S1, S2, S5b,c, S8, S9
BICDR1-mNeonGreen	pOmniBac	This study	F4a-d
LIS1-SNAPf	pOmniBac	Elshenawy et al., 2020[16]	F1d-g, F2, F3, F4, F5b-d, F6, S1-7, S9
LIS1$^{39\text{-}410}$-SNAPf	pOmniBac	This study	F3b,e,f, S5d
LIS1$^{83\text{-}410}$-SNAPf	pOmniBac	This study	F3b,d-f, S5d
LIS1$^{R316A, W340A}$-SNAPf	pOmniBac	This study	F3b,c, F6b, S5a-c
NDE1-SNAPf	pOmniBac	This study	F1, F2, F3c-f, F4, F6, S1-6, S9
NDE1$^{1\text{-}190}$-SNAPf	pOmniBac	This study	F2f-h, F3b, F6b,c, S4, S7, S9
Ybbr-NDE1	pOmniBac	This study	F5
Ybbr-NDE1$^{E118A, R129A}$	pOmniBac	This study	F5, S6f
Ybbr-NDE1$^{E118K, R129E}$	pOmniBac	This study	F5, S6f
Ybbr-NDE1^{E47A}	pOmniBac	This study	F5
Ybbr-NDE1^{E47K}	pOmniBac	This study	F5
NDE1$^{E118A, R129A}$-SNAP	pOmniBac	This study	S6a-e
NDE1$^{E118K, R129E}$ -SNAP	pOmniBac	This study	S6a-e
NDE1^{E47A} -SNAP	pOmniBac	This study	F5d, S6a-e
NDE1^{E47K} -SNAP	pOmniBac	This study	S6a-e
PAFAH α2-SNAPf	pOmniBac	This study	F6, S7, S8, S9

Supplementary Table 1. The list of protein constructs used in this study. Dynein chains were codon-optimized for *Spodoptera frugiperda* (SF9) expression and inserted into the pOmniBac backbone. Plasmid containing NDE1 was obtained from Origene. NDE1, LIS1, PAF-AH1B α2, and BICDR1 constructs were cloned into the pOmniBac backbone. Constructs were tagged with an N-terminal 6xHis-ZZ-TEV site for affinity purification and Tev protease cleavage during protein purification. The SNAPf tag was inserted for labeling the proteins with fluorescent dyes or biotin (F: Figure, S: Supplementary Figure).

Figure	Sample	Complex	Expected (kDa)	Measured (kDa)	%
Figure 2b	Lis1	Lis1	133	148 ± 28	88
	Nde1	Nde1	114	113 ± 23	90
	Lis1 and Nde1	*Lis1 or Nde1	114 or 133	135 ± 15	56
		1 Lis1 + 1 Nde1	247	250 ± 26	15
		2 Lis1 + 1 Nde1	380	393 ± 23	8
Figure 2f	Nde1$^{1-190}$	Nde1$^{1-190}$	84	83 ± 22	95
	Lis1 and Nde1$^{1-190}$	Nde1$^{1-190}$	84	85 ± 19	22
		Lis1	133	148 ± 14	16
		1 Lis1 + 1 Nde1$^{1-190}$	217	234 ± 18	44
		2 Lis1 + 1 Nde1$^{1-190}$	340	382 ± 16	11
Figure 3b	Lis1	Lis1	133	148 ± 28	88
	mtLis1	mtLis1	133	140 ± 27	88
	Lis1$^{39-410}$	Lis1$^{39-410}$	63	69 ± 15	93
	Lis1$^{83-410}$	Lis1$^{83-410}$	57	61 ± 13	100
	Nde1$^{1-190}$ and mtLis1	Nde1$^{1-190}$	84	87 ± 18	51
		mtLis1	133	139 ± 21	49
	Lis1$^{83-410}$ and Nde1$^{1-190}$	Lis1$^{83-410}$	57	54 ± 10	75
		Nde1$^{1-190}$	84	84 ± 19	25
Figure 6b	α2	α2	90	94 ± 19	76
	α2 and Lis1	α2	90	91 ± 14	15
		Lis1	133	138 ± 14	21
		α2 + Lis1	223	228 ± 25	47
	α2 and mtLis1	α2	90	88 ± 14	30
		mtLis1	133	139 ± 27	58
	α2 and Nde1$^{1-190}$	*α2 or Nde1$^{1-190}$	84 or 90	90 ± 9	77
	α2, Lis1, and Nde1$^{1-190}$	*α2 or Nde1$^{1-190}$	84 or 90	91 ± 10	43
		Lis1	133	139 ± 12	13
		*α2 + Lis1 or Nde1$^{1-190}$ + Lis1	217 or 223	229 ± 14	29
Supp. Figure 6b	Nde1^{E118A,R129A}	Nde1^{E118A,R129A}	114	113 ± 18	90
	Nde1^{E118K,R129E}	Nde1^{E118K,R129E}	114	117 ± 22	89
	Nde1^{E47A}	Nde1^{E47A}	114	119 ± 19	93
	Nde1^{E47K}	Nde1^{E47K}	114	120 ± 30	92
Supp. Figure 6e	Lis1 and Nde1^{E118A,R129A}	Nde1^{E118A,R129A}	114	113 ±21	54
		Lis1	133	147 ± 12	37
	Lis1 and Nde1^{E118K,R129E}	Nde1^{E118K,R129E}	114	114 ± 21	58
		Lis1	133	149 ± 10	35
	Lis1 and Nde1^{E47A}	*Nde1^{E47A} or Lis1	114 or 133	120 ± 29	66
		1 Lis1 + 1 Nde1^{E47A}	247	255 ± 27	25
		2 Lis1 + 1 Nde1^{E47A}	380	388 ± 35	7
	Lis1 and Nde1^{E47K}	Nde1^{E47A}	114	115 ± 22	54
		Lis1	133	150 ± 9	14
		1 Lis1 + 1 Nde1^{E47K}	247	258 ± 33	24
		2 Lis1 + 1 Nde1^{E47K}	380	400 ± 21	6

Supplementary Table 2. The parameters of a multi-Gaussian fit of mass photometry measurements. Proteins were diluted to 5-20 nM and mixed at equimolar concentrations. Measured mass and percentage represent the center (mean ± s.d.) and the percent area of the corresponding Gaussian peak (*: two close peaks cannot be distinguished). Expected mass

corresponds to the dimeric forms of Lis1, Nde1, and α2 constructs, except the mass of the monomeric form is reported for $Lis1^{39-410}$ and $Lis1^{83-410}$.

2. Supplementary Figures

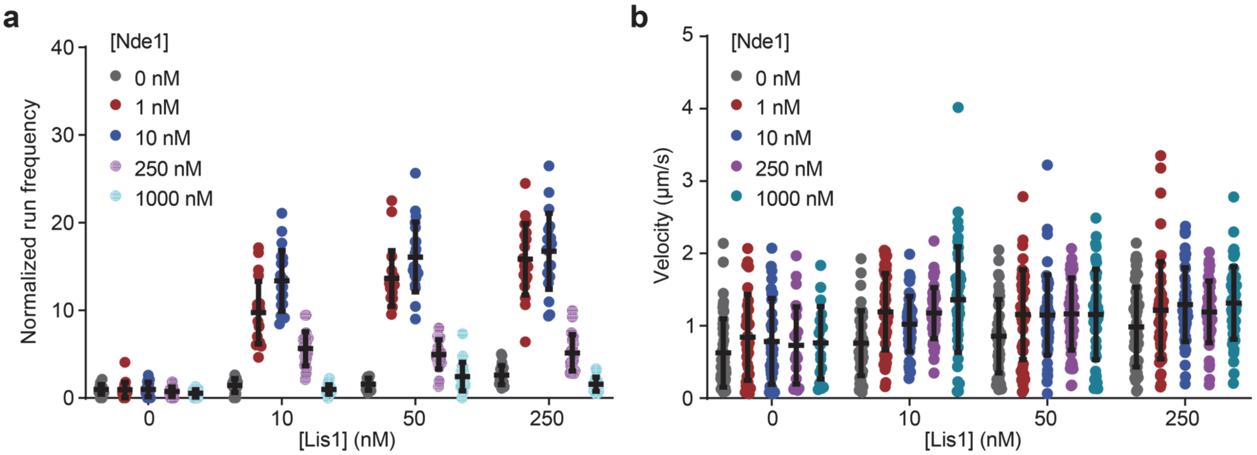

Supplementary Figure 1. Run frequency and velocity of wtDDR under different Lis1 and Nde1 concentrations. a. Normalized run frequency distribution of wtDDR under different Lis1 and Nde1 concentrations. Results were normalized to the 0 nM Lis1 and 0 nM Nde1 condition. The center line and whiskers represent the mean and s.d., respectively. n =20 microtubules for each condition. **b.** Velocity distribution of wtDDR under different Lis1 and Nde1 concentrations. The center line and whiskers represent the mean and s.d., respectively. n =50 processive DDR complexes for each condition. Source data are provided as a Source Data file.

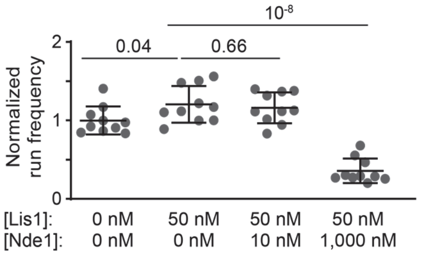

Supplementary Figure 2. mtDDR motility is inhibited by excess Nde1. Normalized run frequencies distribution of mtDDR under different Lis1 and Nde1 concentrations. The center line and whiskers represent the mean and s.d., respectively (n =10 microtubules for each condition). P values are calculated from a two-tailed t-test. Source data are provided as a Source Data file.

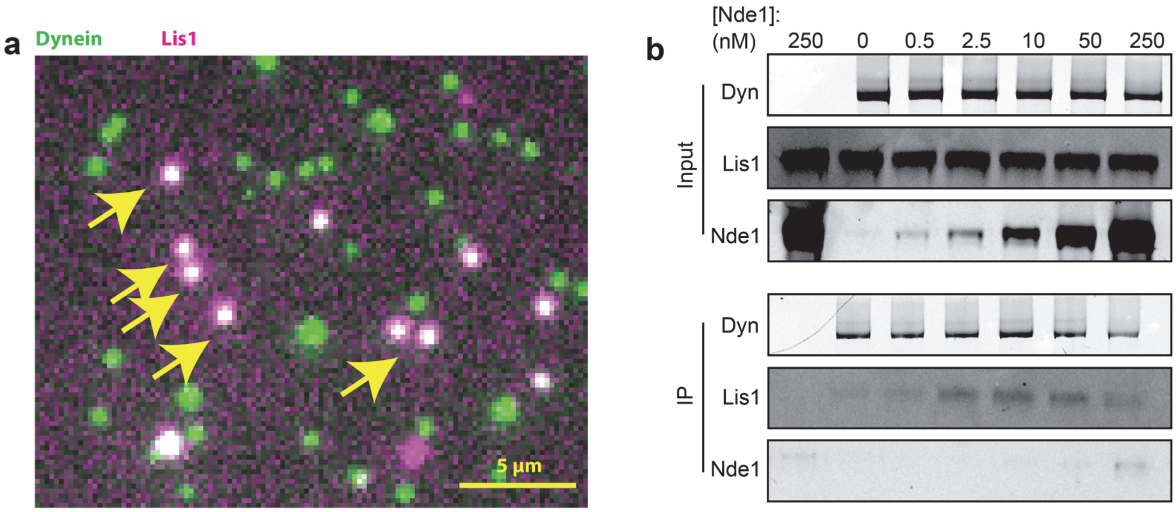

Supplementary Figure 3. Nde1 promotes the association of Lis1 with dynein. a. Representative overlay imaging of surface-immobilized and Alexa488-labeled dynein and Lis1-LD555. Arrows show examples of colocalization between dynein and Lis1. **b.** Co-immunoprecipitation (Co-IP) of Lis1 pulled down by dynein-GFP with increasing Nde1 concentrations. Source data are provided as a Source Data file.

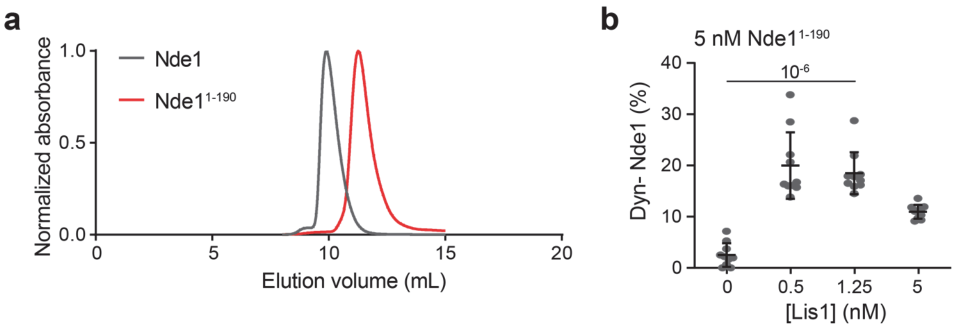

Supplementary Figure 4. Single molecule colocalization assays of Nde1$^{1\text{-}190}$. a. Elution profiles of Nde1 and Nde1$^{1\text{-}190}$ from a size exclusion column. **b.** Percent colocalization of Alexa488-labeled dynein with LD655-labeled Nde1$^{1\text{-}190}$ under increasing concentrations of Lis1 (n =10 imaging areas (40 µm x 40 µm) with at least 100 immobilized dynein spots for each condition). Nde1$^{1\text{-}190}$ concentration was kept at 5 nM. Unlike full-length Nde1, which exhibits higher colocalization with dynein under increasing Lis1 concentrations, Nde1$^{1\text{-}190}$ colocalization to dynein increases at low Lis1 concentrations and decreases at higher Lis1 concentrations. *P* values are calculated from a two-tailed t-test. Source data are provided as a Source Data file.

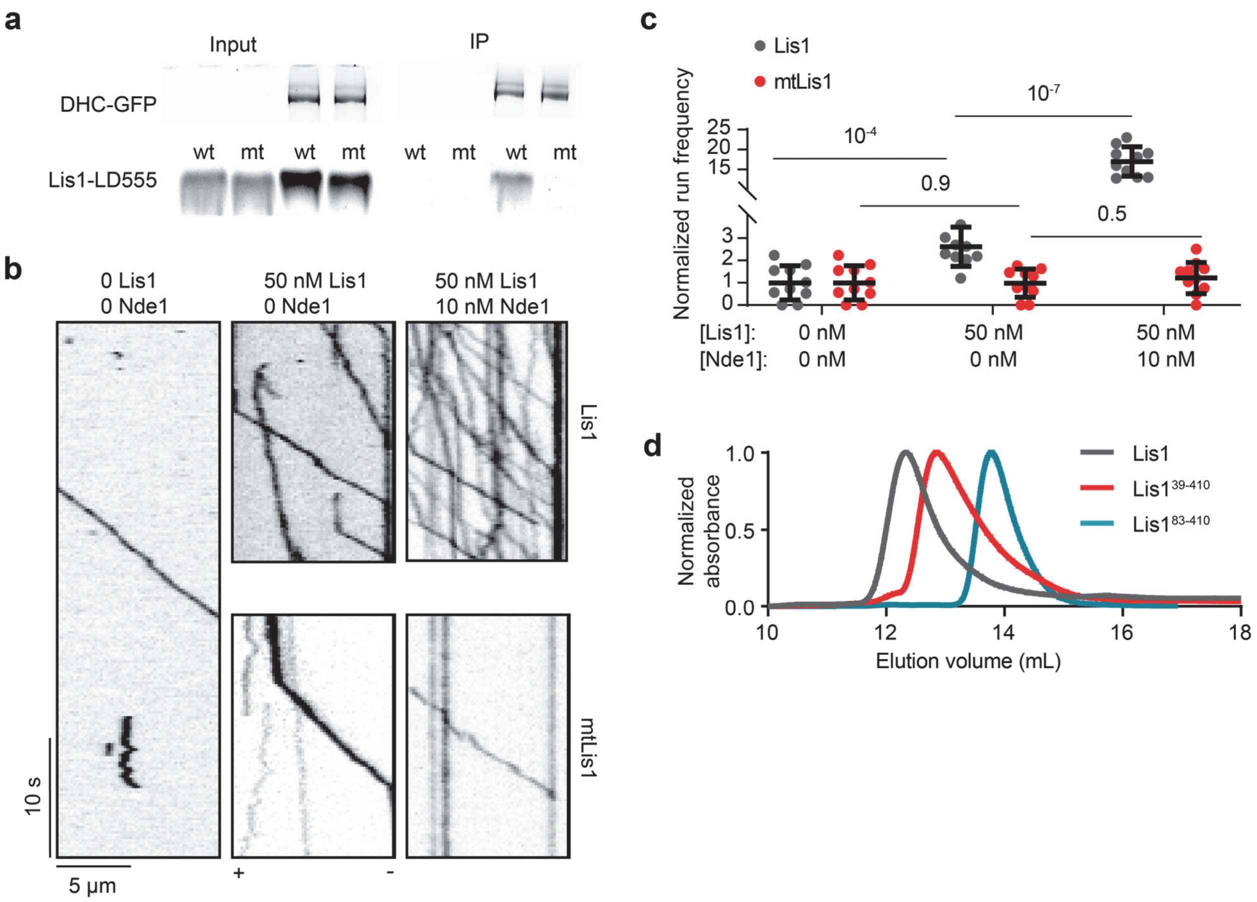

Supplementary Figure 5. Dynein binding mutant of Lis1 does not stimulate dynein activation with or without Nde1. a. Co-IP of Lis1 or mtLis1 with wtDyn-GFP using anti-GFP beads. **b.** Representative kymographs show wtDDR motility with 50 nM Lis1 or mtLis1 with or without 10 nM Nde1. **c.** The run frequency distribution of wtDDR complexes with 50 nM Lis1 or mtLis1 with or without 10 nM Nde1. Results were normalized to the 0 nM Lis1 and 0 nM Nde1 condition. The center line and whiskers represent the mean and s.d., respectively. n =10 microtubules for each condition. P values are calculated from a two-tailed t-test. **d**. Elution profiles of Lis1, Lis1$^{39\text{-}410,}$ and Lis1$^{83\text{-}410}$ from a size exclusion column. Source data are provided as a Source Data file.

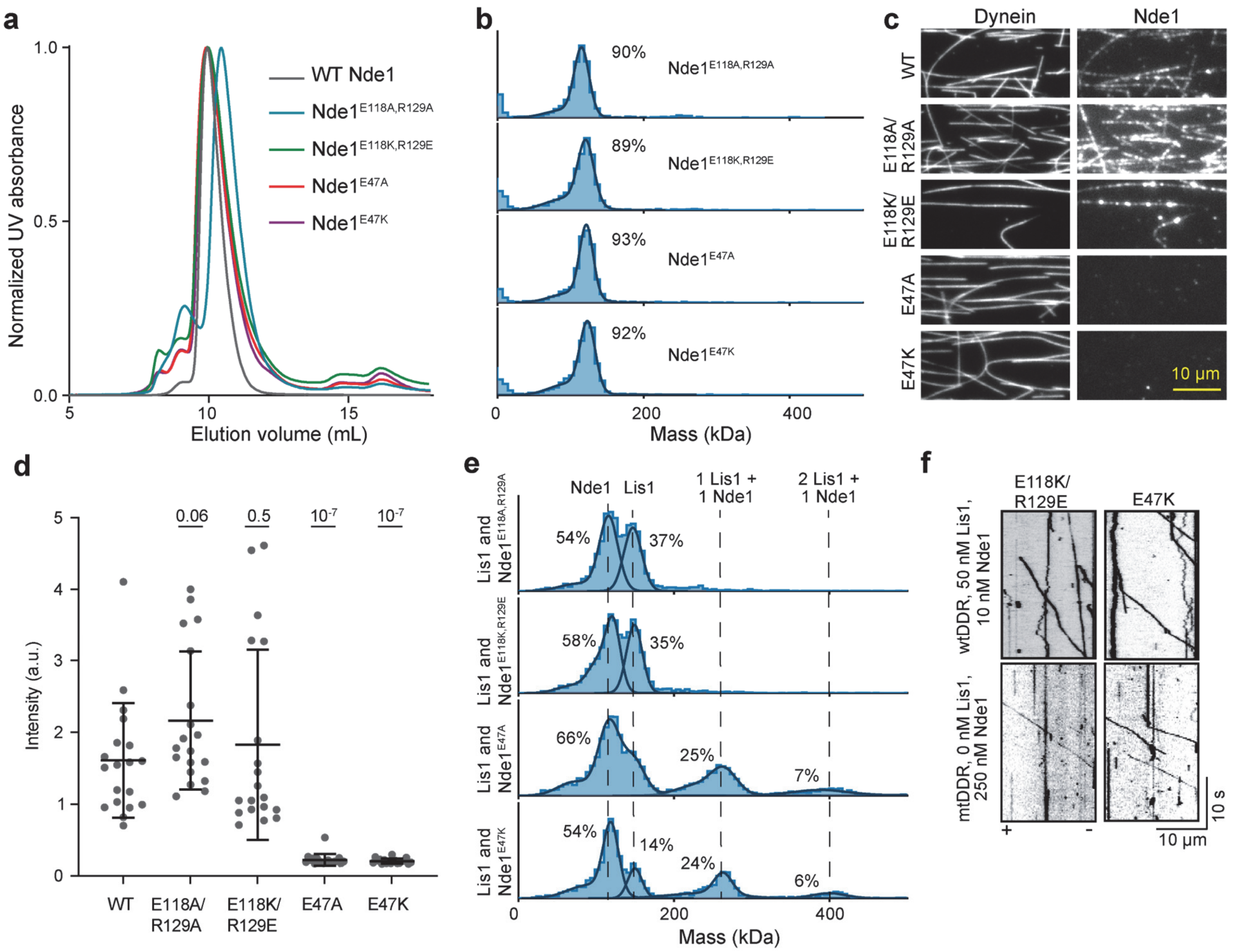

Supplementary Figure 6. Characterization of the Lis1- and dynein-binding mutants of Nde1. a. Elution profiles of wildtype and mutant Nde1 from the SEC column. **b**. Mass photometry shows the Nde1 mutants form dimers. **c**. Representative images of LD655-labeled Nde1 mutants landing on Alexa488-labeled dynein on surface-immobilized microtubules. While Lis1-binding mutants localize to dynein, dynein-binding mutants do not associate with dynein. **d**. The fluorescence intensity of Nde1 mutants landing on Alexa488-labeled dynein on MT. (mean ± s.d.; *n* =19 microtubules for each condition). *P* values are calculated from a two-tailed t-test. **e**. Mass photometry profiles of a mixture of Lis1 and Nde1 mutants. Dynein-binding mutants, but not Lis1-binding mutants, of Nde1 form complexes with Lis1. **f.** Representative kymographs show wtDDR motility with 10 nM charge reversal mutants of Nde1 and 50 nM Lis1 (Top) and mtDDR motility with 250 nM charge reversal mutants of Nde1 in the absence of Lis1 (Bottom). In **b** and **e**, solid curves represent a fit to multiple Gaussians to predict the average mass (Supplementary Table 2) and percentage of each population. Source data are provided as a Source Data file.

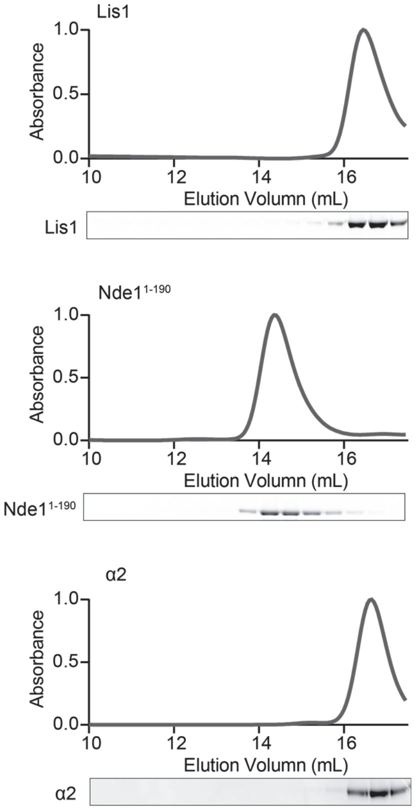

Supplementary Figure 7. Size exclusion profiles of Lis1, Nde1$^{1\text{-}190}$, and α_2. Normalized absorbance (top) and fluorescence picture of a denaturing gel (bottom) of Lis1-LD655, Nde1$^{1\text{-}190}$-LD555, and α_2-Alexa488 eluted from Superose 6 size exclusion column. Although Nde1$^{1\text{-}190}$ and α2 constructs have similar mass, Nde1$^{1\text{-}190}$ elutes first from the column due to its elongated shape.

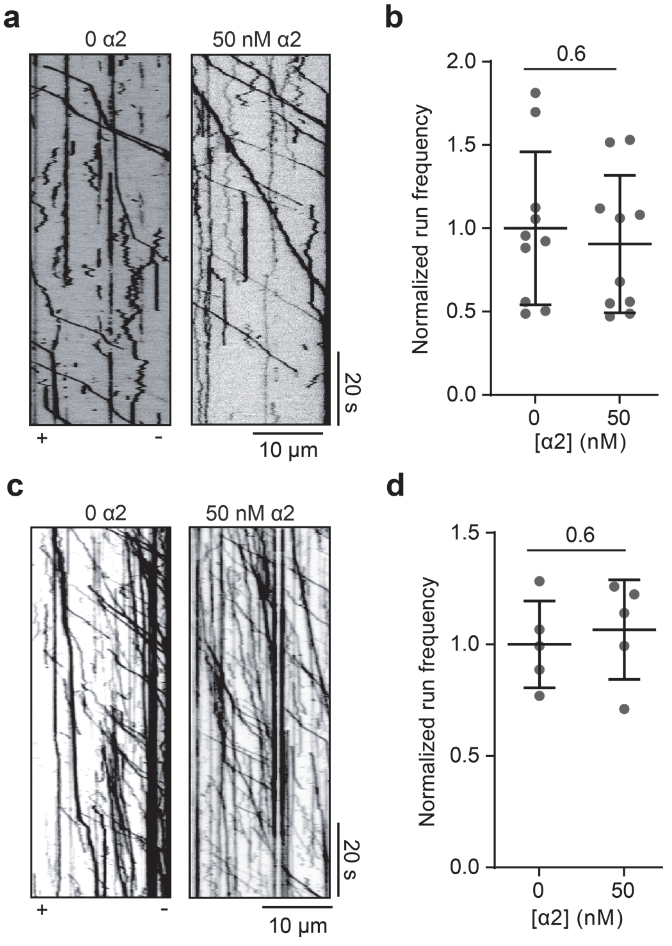

Supplementary Figure 8. α2 does not directly influence DDR motility. a. Representative kymographs show wtDDR motility with or without 50 nM α2. **b.** The run frequency distribution of wtDDR with or without 50 nM α2, respectively. Results were normalized to the 0 nM α2 condition. n =10 microtubules for each condition. **c.** Representative kymographs of the motility of mtDDR complexes with or without α2. **d.** The run frequency distribution of mtDDR with or without 50 nM α2. Results were normalized to the 0 nM α2 condition. n =5 microtubules for each condition. In **b** and **d**, the center line and whiskers represent the mean and s.d., respectively. P values are calculated from a two-tailed t-test. Source data are provided as a Source Data file.

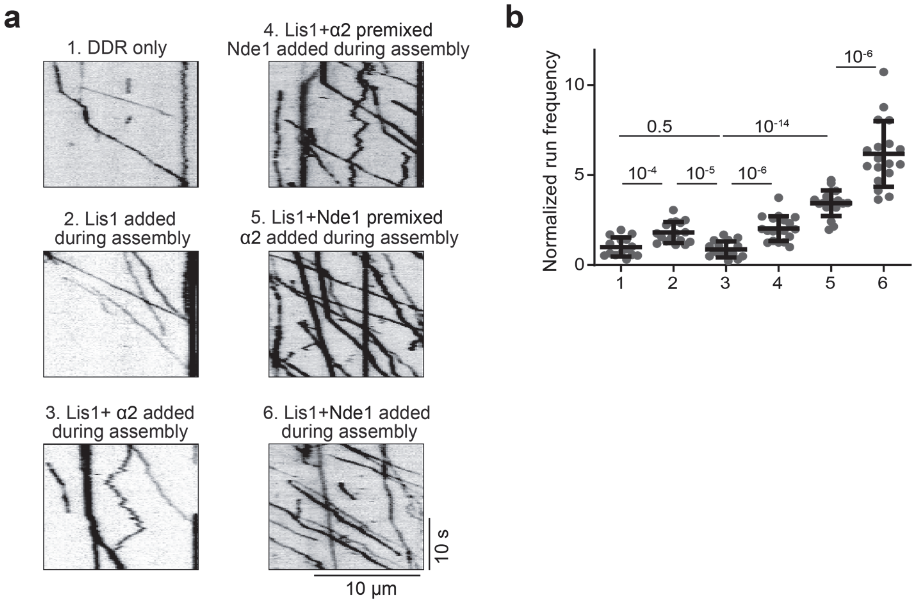

Supplementary Figure 9. Nde1 rescues Lis1-mediated activation of dynein motility in the presence of α2. a. Representative kymographs showing the motility of wtDDR complexes under different wtDDR assembly conditions. The assays were performed in 10 nM wtDyn, 50 nM BicDR1, 150 nM dynactin, 10 nM Lis1, 50 nM Nde1, and 50 nM α2 (DA: during assembly). **b**. The run frequency distribution of wtDDR under different complex assembly conditions shown in a. Results were normalized to the 0 nM α2, 0 nM Lis1, and 0 nM Nde1 condition. From left to right, *n* =15, 17, 16, 19, 19, and 18 microtubules. The center line and whiskers represent the mean and s.d., respectively. *P* values are calculated from a two-tailed t-test. Source data are provided as a Source Data file.

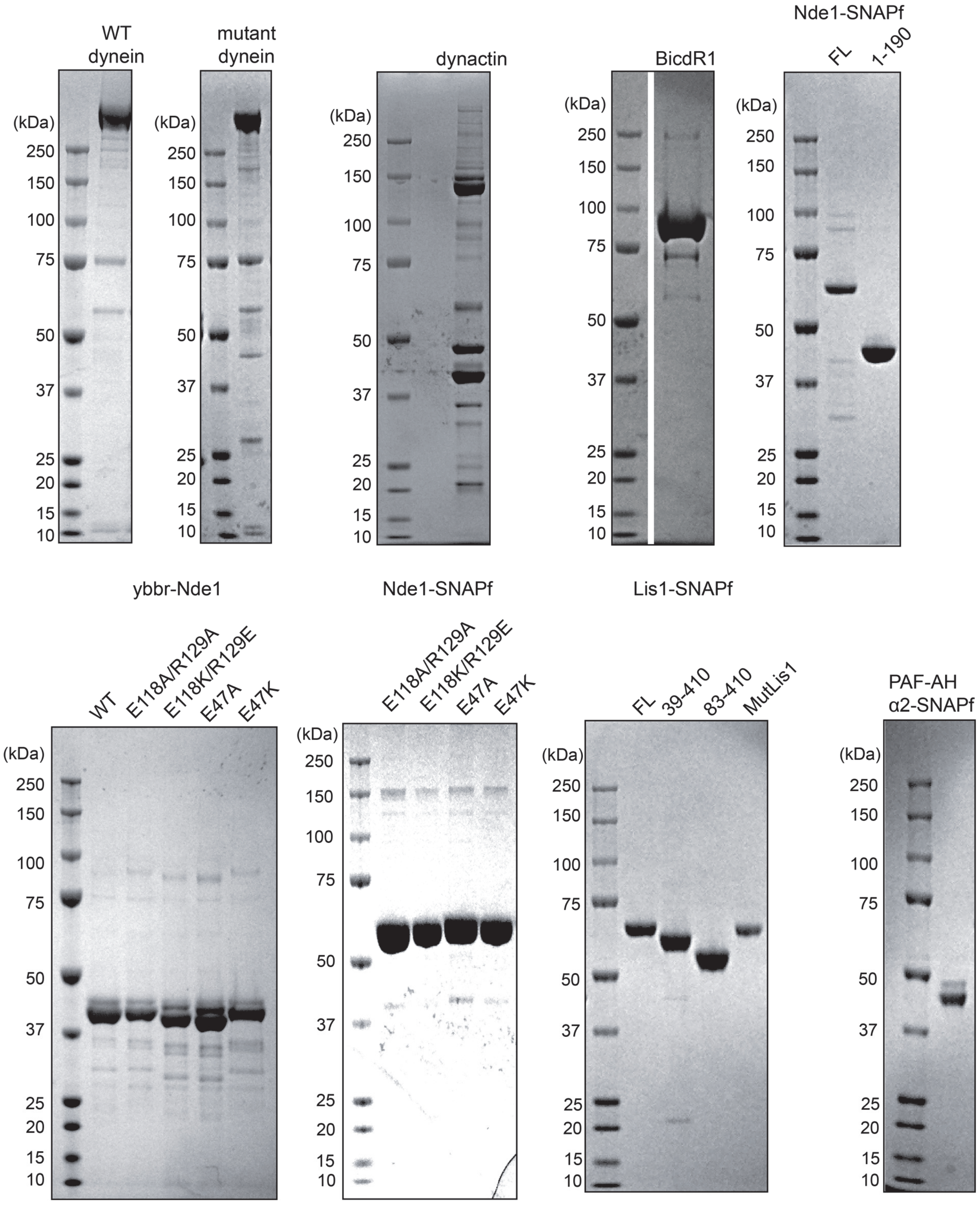

Supplementary Figure 10. Denaturing gel pictures of purified proteins in this study. Source data are provided as a Source Data file.
