# Supplementary material for: Nde1 promotes Lis1-mediated activation of dynein
Source: Nat Commun. 2023 Nov 9;14:7221. doi: 10.1038/s41467-023-42907-x (PMC10632352; doi:10.1038/s41467-023-42907-x)
Supplement: Supplementary file 4 — Description of Additional Supplementary Files [file 41467_2023_42907_MOESM4_ESM.pdf]

Description of Additional Supplementary files

File name: Supplementary Movie 1
Description: wtDDR motility is enhanced by Lis1 and Nde1. Single molecule motility recording of wtDDR complexes in the presence of 0-50nM Lis1 and 0- 1,000 nM Nde1 using a TIRFM assay. The fluorescence signal corresponds to BicDR1-mNeonGreen, while other subunits and factors are unlabeled. Related to Figure 1d.

File name: Supplementary Movie 2
Description: Nde1 does not substantially affect the run frequency of mtDDR. Single molecule motility recording of mtDDR complexes with or without added Lis1 or Nde1 using a TIRFM assay. The fluorescence signal corresponds to BicDR1-mNeonGreen, while other subunits and factors are unlabeled. Related to Figure 1f.

File name: Supplementary Movie 3
Description: AlphaFold2 prediction of Lis1 (purple) binding to Nde1. Related to Figure 2a.

File name: Supplementary Movie 4
Description: wtDDR motility is not enhanced by Nde1 mutants that do not bind either dynein or Lis1. Single molecule motility recording of wtDDR complexes with 50 nM Lis1 and 10 nM Nde1 or different Nde1 mutants using a TIRFM assay. The fluorescence signal corresponds to BicDR1-mNeonGreen, while other subunits and factors are unlabeled. Related to Figure 5b.
